# Basal and LPS-stimulated inflammatory markers and the course of depression and anxiety symptoms

**DOI:** 10.1192/j.eurpsy.2023.1265

**Published:** 2023-07-19

**Authors:** W. A. Van Eeden

**Affiliations:** Psychiatry, Leids Universitair Medisch Centrum, Leiden, Netherlands

## Abstract

**Introduction:**

Multiple studies show an association between inflammation –characterized by increased blood levels of C-reactive protein (CRP) and pro-inflammatory cytokines– and major depressive disorder (MDD). People with chronic low-grade inflammation may be at an increased risk of MDD, often in the form of sickness behaviors. A cross-sectional relationship between low-grade inflammation and anxiety has also been reported, but the potential longitudinal relationship has been less well studied.

**Objectives:**

We aimed to examine whether basal and lipopolysaccharide (LPS-)induced levels of inflammatory markers are associated with depressive and anxiety symptom severity over the course of nine years. We hypothesized that inflammation is predictive of the severity and the course of a subset of symptoms, especially symptoms that overlap with sickness behavior, such as anhedonia, anorexia, low concentration, low energy, loss of libido, psychomotor slowness, irritability, and malaise.

**Methods:**

We tested the association between basal and lipopolysaccharide (LPS)-induced inflammatory markers with individual depressive symptoms (measured using the Inventory of Depressive Symptomatology Self-Report) and anxiety symptoms (measured with the Beck’s Anxiety Inventory; BAI, Fear Questionnaire;FQ and Penn’s State Worry Questionnaire; PSWQ) over a period of up to 9 years using multivariate-adjusted mixed models in 1147 to 2872 Netherlands Study of Depression and Anxiety (NESDA) participants.

**Results:**

At baseline, participants were on average 42.2 years old, 66.5% were women, and 53.9% had a current mood or anxiety disorder. We found that basal and LPS-stimulated inflammatory markers were more strongly associated with sickness behavior symptoms at up to 9-year follow up compared to non-sickness behavior symptoms of depression. However, we also found significant associations with some symptoms that are not typical of sickness behavior (e.g., sympathetic arousal among others). The associations between inflammation and anxiety symptoms were attenuated by 25%-30% after adjusting for the presence of (comorbid) major depressive disorder (MDD), but remained statistically significant.

**Conclusions:**

Inflammation was not related to depression as a unified syndrome but rather to the presence and the course of specific MDD symptoms, of which the majority were related to sickness behavior. With regard to anxiety symptoms, we found that participants with high levels of inflammatory markers have on average high levels of anxiety consisting of physical arousal and agoraphobia, which tended to persist over a period of nine years, albeit with small effect sizes. These associations were partly driven by co-morbid depression. Anti-inflammatory strategies should be tested in the subgroup of MDD patients who report depressive symptoms related to sickness behavior.

**Disclosure of Interest:**

None Declared

